# A Convenient and Simple Ionic Polymer-Metal Composite (IPMC) Actuator Based on a Platinum-Coated Sulfonated Poly(ether ether ketone)–Polyaniline Composite Membrane

**DOI:** 10.3390/polym14040668

**Published:** 2022-02-10

**Authors:** Mohammad Luqman, Hamid M. Shaikh, Arfat Anis, Saeed M. Al-Zahrani, Mohammad Asif Alam

**Affiliations:** 1 Department of Chemical Engineering, College of Engineering, Taibah University, Yanbu 46412, Saudi Arabia; 2 SABIC Polymer Research Centre, Department of Chemical Engineering, King Saud University, P.O. Box 800, Riyadh 11421, Saudi Arabia; aarfat@ksu.edu.sa (A.A.); szahrani@ksu.edu.sa (S.M.A.-Z.); 3 Center of Excellence for Research in Engineering Materials (CEREM), King Saud University, P.O. Box 800, Riyadh 11421, Saudi Arabia; moalam@ksu.edu.sa

**Keywords:** ionic polymer-metal composite (IPMC), sulfonated polyether ether ketone (SPEEK) polyaniline (PANI) membrane, actuator, platinum-coated, composite, proton exchange membrane, robotics, biomimetic

## Abstract

Herein, we present new approaches for developing sulfonated polyether ether ketone (SPEEK) and polyaniline-based (PANI) actuator formed by film-casting and chemical reduction of Pt electrodes. We have thoroughly studied the synthesis of SPEEK and characterized it by different analytical techniques. The ion-exchange capacity (IEC) and proton conductivity of SPEEK-PANI polymer membrane were calculated to be 1.98 mmol g^−1^ and 1.97 × 10^−3^ S cm^−1^, respectively. To develop an IPMC actuator, SPEEK was combined with PANI through in-situ polymerization method. SEM and XRD were used to check the morphology of the given SPEEK-PANI-Pt membrane. In addition, FT-IR and EDX techniques confirmed the molecular structure and chemical conformation of SPEEK-PANI polymer membrane. Pt electrode layers homogeneously dispersed on the IPMC membrane surface, which was demonstrated by smooth SEM micrographs. The actuation functioning, including the high bending deflection, proton conductivity, current density and IEC of IPMC actuator based on SPEEK-PANI-Pt, was obtained owing to its strong electrochemical and electromechanical characteristics. Synergistic combinations of SPEEK and PANI produced membrane that are flexible, mechanically strong and robust. The developed materials have immense capability as actuators for various applications including in biomimetics and robotics.

## 1. Introduction

The smart materials such as electroactive polymers (EAPs) that respond to electrical stimulation [1], including conducting polymers (CPs) [2], ionic gels [3], solid electrolyte composite comprising ionic liquids [4] and ion-exchange membrane [5], may be used to facilitate ion migration within a polymer matrix. The ionic polymer-metal composites (IPMCs) based EAPs, which have the advantages of lightweight, easy synthesis, and adaptability in harsh and varying environments [2], are frequently called “artificial muscles” and are being developed as favorable materials in the field of the naturally animating robots and biomedical frameworks [6,7,8,9,10,11,12,13,14,15,16,17,18,19]. IPMC-based actuators can be customized as needed based on the electrochemical characteristics of polymer materials. The conventional IPMCs are made from a composite membrane or ion-exchange polymer with novel metal electrodes plated on top and having an inner medium of solvent. IPMC membranes actuate due to an electric field produced by a voltage difference between electrodes. The literature review suggests that a number of commercially available per-fluorinated polymers, including Nafion^®^ (DuPont, Wilmington, NC, USA) and Aciplex^®^ (Asahi Chemical, Osaka, Japan), are mainly being used for many IPMC applications. This may be, most probably, owing to their high proton conductivity, excellent chemical stability, and mechanical strength. However, a very high cost, a low generative blocking force and a short operation time in addition to a few other factors [7] of these traditional IPMCs have led researchers to find cost-effective, high-performing, and environmentally more friendly alternatives to these IPMCs.

IPMCs have been proving their potential use in robotics because of their capability to exhibit bio-inspired flexibility and considerable deflection at a modest operating voltage, converse to traditional actuators such as motors [20,21]. In order to establish IPMC as a reliable actuator component in soft robotics, an efficient IPMC actuator is essential for delivering appropriate force response and high and fast deflections.

In view of the above discussion, a novel and effective actuator based on ionic aromatic polyether ether ketone (PEEK) was developed. The PEEK has a non-fluorinated aromatic backbone, where 1,4-disubstituted phenyl groups are parted by carbonyl (-CO-) and ether (-O-) bonds. The semicrystalline PEEK has exceptional mechanical properties, chemical resistance, thermal stability, and toughness. It is an appropriate polymer for many industries including medical, aerospace, microfiltration (MF), electronic industries, ion-conducting membranes, reverse osmosis (RO), and ultrafiltration (UF). The sulfonation process is a useful polymer variation method that is particularly appropriate for aromatic polymers. An aromatic PEEK is sulfonated to increase hydrophilicity and acidity, since water enables the transfer of protons and improves the ionic conductivity. The SPEEK is a copolymer having sulfonated PEEK (hydrophilic units) and non-sulfonated PEEK (hydrophobic units) structural units. The sulfonated PEEK (SPEEK) microphase separates itself into hydrophobic and hydrophilic parts. The water absorbed by a hydrophilic part of the polymers associating with the −SO_3_H groups leads to swelling and the conduction of protons [22]. Moreover, SPEEK upon 100% sulfonation, can dissolve in water [23], suggesting a high level of hydrophilicity. The engineering thermoplastic PEEKs have a variety of attractive properties, such as high thermo-oxidative stability and excellent mechanical and solvent resistance properties [24]. It is possible to solfonate PEEKs with a degree of sulfonation of 0.5 to 1.0 per repeat unit. The PEEK can be sulfonated utilizing concentrated sulphuric acid, sulphur trioxide, methane sulfonic acid, chlorosulfonic acid, and acetyl sulphate [25,26,27,28,29,30]. Various sulfonated PEEK polymers can be synthesized as free acids (-SO_3_H), esters (-SO_2_R), salts (-SO_3_^−^Na^+^), and different derivatives. The decrease or disorder in the ether group content of polymer chains (PEKEKK > PEEKK/PEK > PEEK) adversely affects sulfonation [31].

**Figure 1:**
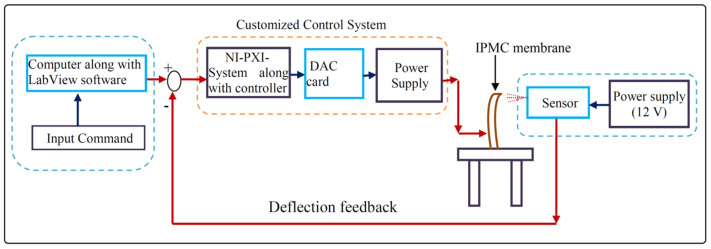
Shows the schematic diagram for actuation and control of SPEEK-PANI-Pt ionic polymer actuator.

An IPMC membrane with SPEEK-polyaniline (PANI) polymer composite was developed to analyze the effectiveness of actuators. The redox cyclability and conducting nature of PANI increased the bending and displacement rate while reducing the back-relaxation phenomenon. This research will pave the way for a new generation of IPMC actuators with a SPEEK-PANI-Pt that can deflect large deflections for the development of microrobotics. There were two important objectives of this study: (1) Development of an innovative microgripping system based on SPEEK-PANI IPMC membrane and Pt electrode (SPEEK-PANI-Pt); and (2) Characterization and demonstration of SPEEK-PANI-Pt actuator. This study also offers other significant advantages, including lightweight material, a low operating voltage (0–5.25 V), availability of the membrane in numerous shapes and sizes, and the necessity to develop microrobots utilizing a simple controller. Figure 1 demonstrates the schematic diagram of the experimental setup, which is based on the determination of bending behavior of the IPMC (SPEEK-PANI-Pt) actuator corresponding to voltage.

## 2. Materials and Methods

### 2.1. Materials Used in This Study

Aniline (C_6_H_5_NH_2_) was purchased from Central Drug House, (Mumbai, India) Pvt. Ltd. Potassium peroxodisulfate (K_2_S_2_O_8_) and ammonium hydroxide (25%; NH_4_OH) were acquired from Merck Specialties Pvt., Ltd., Darmstadt, Germany. Sodium borohydride (NaBH_4_) and Pt(NH_3_)_4_Cl_2_·H_2_O (crystalline) were purchased from Alfa Aesar, Tewksbury USA. Poly(ether ether ketone) (PEEK) (Vitrex 450PF) was received from Victrex, Lancashire, UK. Concentrated sulfuric acid (95~98%) was obtained from Central drug house (Mumbai, India. All materials were used as received.

### 2.2. Preparation of the Reagent Solutions

10% *v*/*v* aniline’s solution and 0.1 M potassium peroxodisulfate solution were prepared in 1 M HCl solution. Demineralized water was used for the preparation of NaBH_4_ (5.0%), NH_4_OH (5.0%), and Pt(NH_3_)_4_Cl_2_·H_2_O (0.04 M) solutions.

### 2.3. Preparation of SPEEK Membrane

The SPEEK membrane was synthesized according to the given reference [29]. Briefly, 500 mL sulphuric acid (concentrated) was added in 10.0 g of PEEK and stirred for 18 h at 25 °C. Then, the highly acidic mixture was slowly poured into ice-cooled deionized water under continuous and vigorous stirring. Hence, the sulfonation reaction was interrupted, resulting in the precipitation of the SPEEK polymer as a fine suspension. After that, the washing of the precipitated polymer was carried out by deionized water (DI) several times until the pH became neutral. The polymer was dried in a convection oven at 60 °C for 24 h and then in a vacuum oven for the same period [32,33,34,35,36,37].

### 2.4. Fabrication of SPEEK-PANI Membrane

The fabricated SPEEK membrane was cast into a petri dish and covered by filter paper (Whatman, No. 1). Then, it was kept in a thermostat oven set to 45 °C. The membrane was crosslinked by keeping it in a thermostat oven at 150 °C for 1 h. After drying, 50 mL of aniline solution was added into the membrane; subsequently, 60 mL of K_2_S_2_O_8_ solution was poured dropwise into it. The in situ polymerization of aniline on the SPEEK polymer membrane was carried out with constant stirring for 0.5 h at a temperature not exceeding 10 °C. After that, the mixture was covered by aluminum foil and kept for digestion (24 h) in a refrigerator.

### 2.5. Proton Conductivity and Ion-Exchange Capacity

The studies of the fabricated membrane (SPEEK–PANI), such as proton conductivity and ion-exchange capacity, were calculated as reported by Inamuddin et al. [8].

#### 2.5.1. Proton Conductivity

Proton conductivity of SPEEK–PANI membrane (1 cm × 3 cm) was calculated as follows. Briefly, the impedance analyzer (FRA32M.X) that was connected to Autolab 302N modular potentiostat/galvanostat worked over a frequency of 100 kHz and an AC perturbation of 10 mV was applied to the cell. Initially, the membrane was placed in demineralized water overnight at room temperature. The Equation (1) was used for calculating the proton conductivity (σ, 1.97 × 10^−3^ S cm^−1^)

(1) $$\sigma =\frac{L}{R\times A}$$

#### 2.5.2. Ion-Exchange Capacity

The ion-exchange capacity of the SPEEK–PANI was determined by immerging the membrane in saturated NaCl solution (50 mL) for a day at room temperature. Then, to ensure that all the H^+^ (protons) within the membrane were ion-exchanged with Na^+^, once again the membrane was dipped in a freshly prepared sodium chloride solution and kept for 12 h. The membrane was taken out of the solution, and then the obtained acidic solutions were mixed. Following this, the solution given is titrated by 0.1 M NaOH using a phenolphthalein indicator. The IEC of the membrane was calculated to be 1.98 mmol g^−1^ using Equation (2).

(2) $$IEC=\frac{\mathrm{CV}}{W_{dry}}$$

where the symbols have a specific meaning, C is the NaOH solution’s concentration, V is the volume of NaOH solution consumed, and *W_dry_* is the dry weight of the membrane sample in H^+^ form

### 2.6. Chemical Plating

The Pt metal coating on the fabricated membrane (SPEEK–PANI) was carried out by standard electroless plating protocol. Firstly, both sides of the fabricated membrane were roughened by sandpaper and then cleaned for 20 min using an ultra-sonicator. The membrane was placed in HCl solution (2 M) and then neutralized by double distilled water (DDW). The membrane was coated by simultaneously adding 4.5 mL of Pt(NH_3_)_4_Cl_2_·H_2_O and 1 mL of NH_4_OH aqueous solutions. The digestion of the membrane was completed by keeping it for 6 h. From the membrane’s surface, the excess Pt ions were removed by washing with DDW. 1 mL of NaBH_4_ was added 5 to 6 times every 20 min for the reduction of Pt ions into Pt metal. Then, 5 mL of NaBH_4_ was mixed into it using stirrer. After being washed by DDW, the reaction was terminated by immersing membrane into an HCl (0.1 M) solution. The water molecules surrounded the materials and once the electric field was applied, all the cations diffused towards it. Anions were linked as clusters inside the polymer framework, creating routes for cations to move toward the electrode. This movement of ions led to the bending of the structure in the direction of the anode.

### 2.7. FTIR Analysis

Attenuated total reflectance-Fourier transform infrared spectroscopy (ATR-FTIR) analysis was carried out using a Nicolet iN10 FTIR microscope (Thermo Scientific, Winsford, UK) with a Germanium microtip to determine the functional groups.

### 2.8. Morphological Analysis

Scanning electron microscopy (SEM, JSM-6360A, JEOL, Tokyo, Japan) was used to examine the surface morphology. The thin membrane was placed on conducting carbon tape for analysis. The accelerating voltage was kept at 5 kV, and all of the samples were gold-sputtered.

### 2.9. Wide-Angle X-ray Diffraction (WAXRD)

The membrane samples were examined using wide-angle X-ray diffraction (XRD). A computer-controlled wide-angle goniometer coupled to a sealed-tube source of Cu-Kα radiation (λ = 1.54056 Å) was used. All samples were scanned at 5°/min and 20 ranged from 5 to 80°.

## 3. Results and Discussion

### 3.1. Fourier Transform Infrared (FT-IR) Spectroscopic Measurements

**Figure 2:**
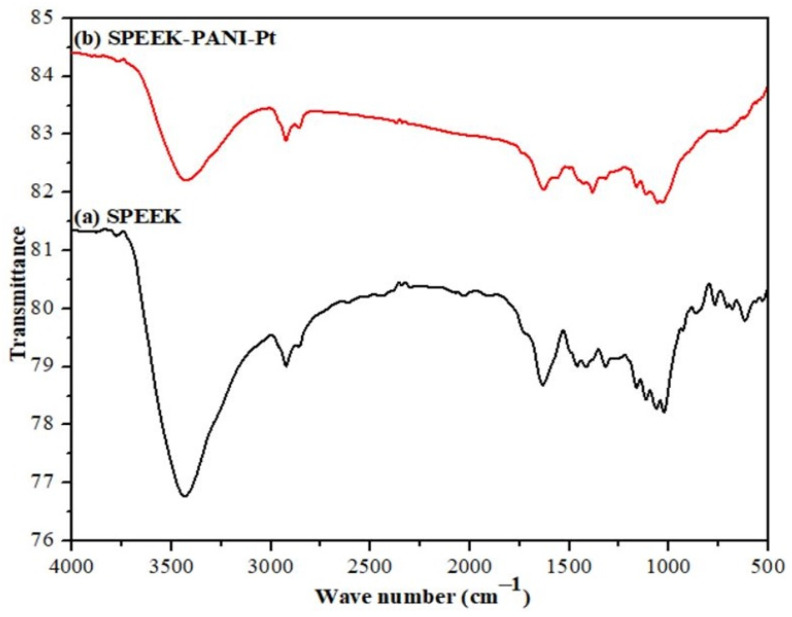
FTIR spectrum of (**a**) SPEEK (**b**) SPEEK-PANI-Pt membrane.

FT-IR spectroscopic studies of SPEEK and SPEEK-PANI polymer membranes were used to confirm that the sulfonic acid group was successfully introduced to PEEK and that the PANI was adequately functionalized. It can be seen from Figure 2a that the absorption peak of -OH group is arisen at 3434 cm^−1^ due to the presence of moisture and -SO_3_H functional group present in the SPEEK.

The two characteristic absorption bands appeared at 1034 and 1089 cm^−1^, signifying the presence of S=O and O=S=O stretches (-SO_3_H functional group) in the characterized moiety. As shown in Figure 2b, the absorption peak of C-O stretch of SPEEK is obtained at 1210 cm^−1^. The C=C group of PANI is observed at 1580 cm^−1^ and C=O streching at 1720 cm-^1^ is due to the carbonyl group of SPEEK membrane. The formation of SPEEK-PANI-Pt polymer membrane was confirmed by the two absorption bands, which are at 1378 and 1574 cm^−^^1^ in Figure 2b. The band at 1378 cm^−^^1^ corresponds to C-N stretching vibrations of the benzene ring., whereas the latter absorption band corresponds to the C=C stretching vibrations of the quinonoid and benzenoid rings, respectively [32,33,34,35,36,37].

### 3.2. X-ray Diffraction (XRD) Studies

**Figure 3:**
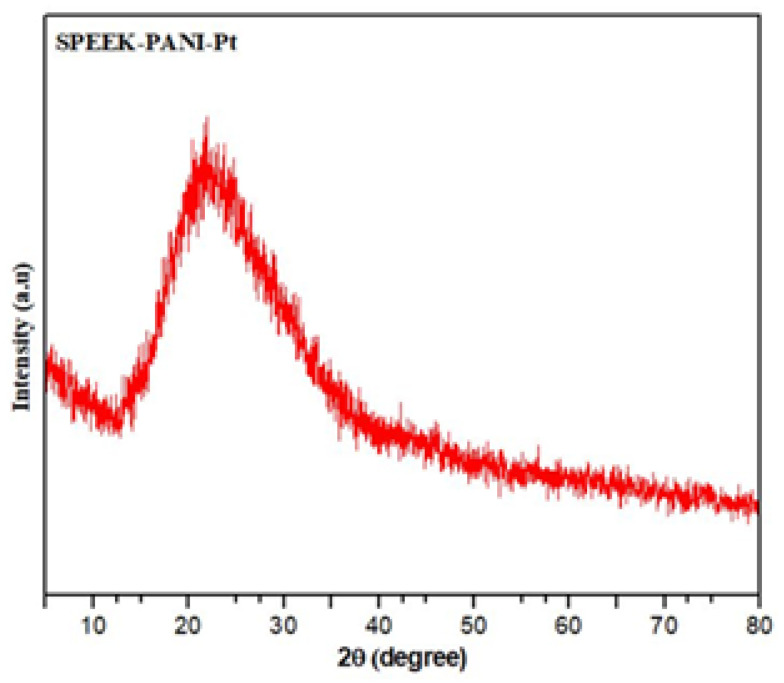
Powder x-ray diffraction pattern of SPEEK-PANI-Pt membrane.

The XRD spectrum of SPEEK-PANI-Pt polymer membrane actuator is shown in Figure 3. In general, the crystallized and amorphous polymer membranes correspondingly produce sharp (high intensity) and broad (low intensity) peaks [35].

It can be seen from Figure 3 that the SPEEK-PANI-Pt polymer membrane exhibits a relatively small peak of 20 values in its X-ray diffraction pattern, which revealed that SPEEK-PANI-Pt is amorphous in nature.

### 3.3. SEM and EDX Studies

**Figure 4:**
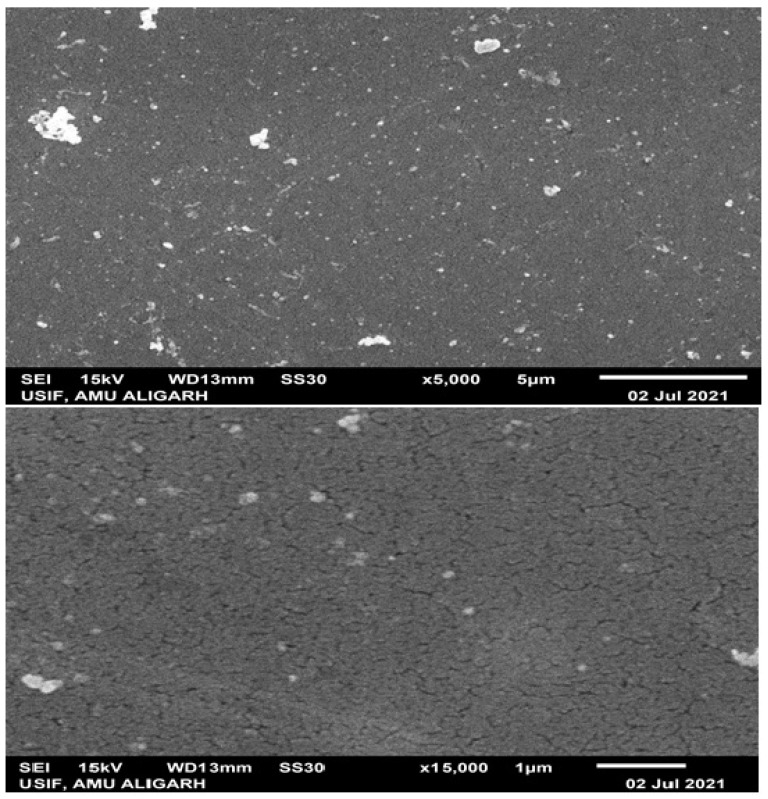
SEM micrographs of SPEEK-PANI-Pt membranes at different magnifications (**top**) 5000× (**down**) 15,000×.

The cross-sectional and surface morphologies of IPMC actuators based on SPEEK-PANI-Pt are depicted in Figure 4 (top, down). It shows that the Pt particles were uniformly distributed within the surface of the SPEEK-PANI membrane, covering the entire IPMC interface.

**Figure 5:**
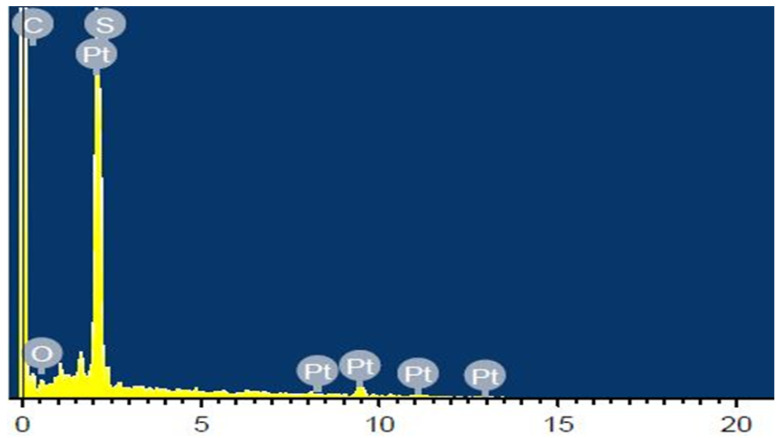
EDX spectrum of SPEEK-PANI-Pt membrane.

The interfacial adhesion among the polymer membrane and electrode can be improved in this IPMC actuator by using the diffusion layers of the Pt. Furthermore, the composition of the acquired IPMC actuator based on the SPEEK-PANI-Pt membrane was confirmed by EDX analysis. The outcomes of EDX measurements were portrayed in Figure 5. It can be seen from the figure the characteristic peaks of the elements, such as platinum, sulphur, carbon, and oxygen, were observed in the EDX spectrum of the concerned membrane surface. The homogeneously and exceptional coating of the Pt electrode on the IPMC actuator surface based on SPEEK-PANI-Pt is confirmed by the prominent amount of the Pt on the surface, indicating excellent performance of the analyzed actuator.

### 3.4. Electro-Mechanical Characterizations

To evaluate the electromechanical behavior of the SPEEK-PANI-Pt ionic polymer film actuator, an experimental testing setup was developed as shown in Figure 1.

**Figure 6:**
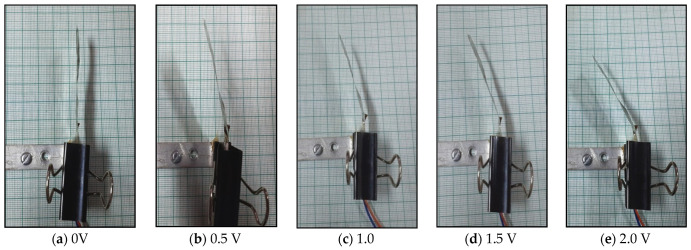

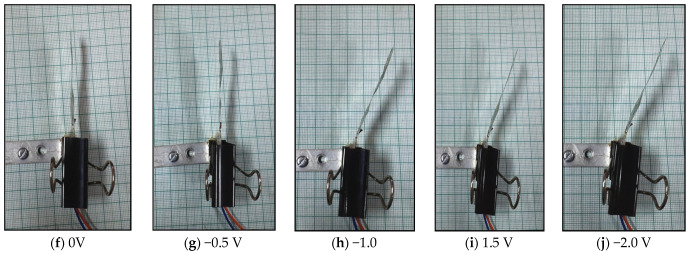
Successive bi-directional deflection behavior of SPEEK-PANI-Pt ionic polymer film actuator.

The SPEEK-PANI-Pt ionic polymer actuator is held in a cantilever arrangement connected to the digital power supply and digital-analog-card (DAC) and NI-PXI system along with microcontroller. For controlling the voltage of the SPEEK-PANI-Pt ionic polymer actuator, a Lab View software was used where input command is sent through designed VI. A laser displacement sensor is used for providing the displacement feedback during measuring the tip displacement of the ionic polymer actuator under-voltage condition of ±3 V DC. For conversion of data, RS-485 to RS-232 serial communication standard protocol was implemented with NI-PXI system, which accomplishes the proper communication between input command and sensor. The experiments were conducted, and successive bending responses are shown in Figure 6.

The tip deflection data of the membrane actuators with sizing of 45 mm × 14 mm × 0.2 mm is given in Table 1.

**Figure 7:**
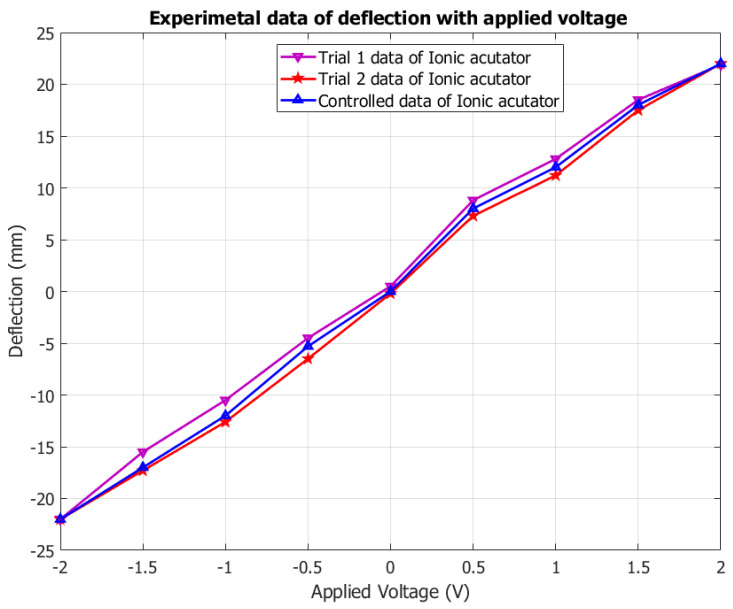
Experimental performance of deflection and voltage for developed SPEEK-PANI-Pt ionic polymer film actuator.

After plotting the behavior (Figure 7), it is found that the deflection behavior of the fabricated membrane actuator shows hysteresis. After repeating experiments, the hysteresis curve reveals that when the voltage increases, the SPEEK-PANI-Pt ionic polymer actuator increases in tip deflection while reducing the voltage from maximum to minimum, and the ionic polymer actuator did not attend the same behavior and provides some deflection error (hysteresis). This hysteresis is minimized using proportional integral derivative (PID) control during the implementation of controlling the actuation behavior, as shown in Figure 7.

**Figure 8:**
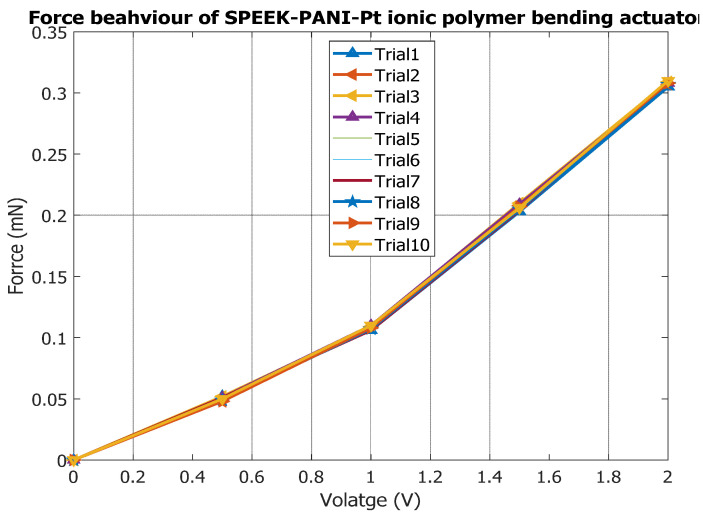
Force behavior of SPEEK-PANI-Pt ionic polymer bending actuator.

To analyse the actuation force characterization of the SPEEK-PANI-Pt ionic polymer actuator, a mini-load cell was used. The SPEEK-PANI-Pt ionic polymer actuator was held in a cantilever arrangement, and the tip of the actuator touches the pan of the mini-load cell. While applying controlled voltage, the different trials are conducted with SPEEK-PANI-Pt ionic polymer bending actuator and experimental data are summarized in Table 2. From experimental data, it is found that the maximum load which could be produced by this bending actuator is 0.31 mN, as shown in Figure 8.

**Figure 9:**
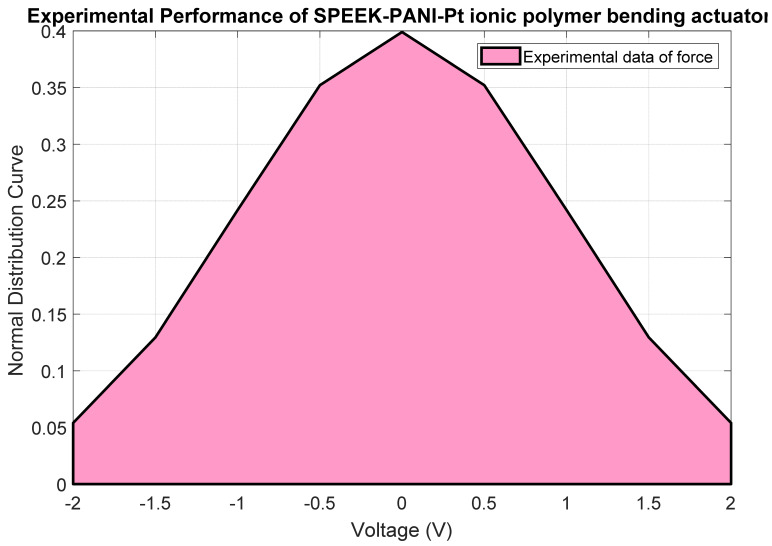
Normal distribution behavior of SPEEK-PANI-Pt ionic polymer bending actuator.

The normal distribution curve for the SPEEK-PANI-Pt ionic polymer bending actuator is plotted, as shown in Figure 9. The shape of the distribution reveals that the error of the SPEEK-PANI-Pt ionic polymer bending actuator has been minimized and shows good repeatability of the forced behavior and repeatedly of this actuator is 88.94%.

## 4. Conclusions

In this work, SPEEK–PANI–Pt membrane was developed using an electroless plating procedure, with the aim for it to be utilized in microrobotic applications. The fabricated membrane showed a high ion exchange capacity, good proton conductivity, and faster actuation capability. Additionally, the large water-holding capacity and low water loss (49% at 6 V) was observed. The linear relation between the voltage and deflection was also observed up to 1.8 V. The membrane showed load-carrying capacity of 0.08 g with a compliant microgripping. Therefore, the synthesized membrane could be effectively used for actuation purposes, which will open a new avenue for the rapidly growing field of microrobotics.

## Figures and Tables

**Table 1:** Experimental deflection data of SPEEK-PANI-Pt ionic polymer actuator.

Tip Deflection (mm)					
Voltage	0 V	0.5V	1.0V	1.5V	2.0V
Trial 1	0	8.0	12.0	18.0	21.0
Trial 2	0	7.3	12.8	17.8	21.9
Trial 3	0	7.5	12.5	17.5	21.5
Trial 4	0	7.6	12.8	17.9	21.2
Trial 5	0	7.9	12.2	17.8	21.7
Trial 6	0	8.0	12.4	18.1	21.8
Trial 7	0	7.8	12.1	17.9	21.5
Trial 8	0	7.7	12.3	18.1	21.8
Trial 9	0	7.8	12.1	17.8	21.7
Trial 10	0	7.9	12.4	18.0	21.9

**Table 2:** Force data of SPEEK-PANI-Pt ionic polymer bending actuator.

Voltage (V)	F_1_ (mN)	F_2_ (mN)	F_3_ (mN)	F_4_ (mN)	F_5_ (mN)	F_6_ (mN)	F_7_ (mN)	F_8_ (mN)	F_9_ (mN)	F_10_ (mN)	Average Force Value (F) in mN
0	0	0	0	0	0	0	0	0	0	0	0
0.5	0.052	0.048	0.052	0.051	0.049	0.05	0.051	0.049	0.048	0.050	0.05
1.0	0.106	0.108	0.109	0.11	0.107	0.109	0.106	0.107	0.108	0.110	0.108
1.5	0.203	0.208	0.21	0.209	0.205	0.207	0.204	0.205	0.207	0.206	0.2064
2.0	0.305	0.307	0.309	0.308	0.306	0.308	0.309	0.307	0.308	0.310	0.3077
Mean											0.13442
Standard Deviation											0.110544623
Repeatability											88.94%

## Data Availability

Data are contained within the article.
